# Efficacy and safety of dapagliflozin in patients with CKD: real-world experience in 93 Italian renal clinics

**DOI:** 10.1093/ckj/sfae396

**Published:** 2024-12-03

**Authors:** Roberto Minutolo, Silvio Borrelli, Andrea Ambrosini, Luigi Amoroso, Filippo Aucella, Valentina Batini, Yuri Battaglia, Laura Bregoli, Vincenzo Cantaluppi, Giuseppe Cianciolo, Paolo Conti, Paolo Fabbrini, Carlo Giammarresi, Egidio Imbalzano, Sandra La Rosa, Marita Marengo, Vincenzo Montinaro, Dario Musone, Marcello Napoli, Felice Nappi, Corrado Pluvio, Domenico Santoro, Roberto Scarpioni, Franco Sopranzi, Tiziana Tullio, Luca De Nicola

**Affiliations:** Nephrology and Dialysis Unit, Department of Advanced Medical and Surgical Sciences, University of Campania, Naples, Italy; Nephrology and Dialysis Unit, Department of Advanced Medical and Surgical Sciences, University of Campania, Naples, Italy; Nephrology and Dialysis Unit, ASST dei Sette Laghi, Varese, Italy; Nephrology and Dialysis Unit, “G. Mazzini” Hospital, Teramo, Italy; Nephrology and Dialysis Unit, Casa Sollievo della Sofferenza Foundation, San Giovanni Rotondo, Italy; Nephrology Unit ASL Toscana NordOvest, Livorno, Italy; Department of Medicine, University of Verona, Verona, Italy; Nephrology and Dialysis Unit, Pederzoli Hospital, Peschiera del Garda, Italy; Nephrology Unit, ASST Spedali Civili Brescia; Nephrology and Kidney Transplantation Unit, Department of Translational Medicine (DIMET), University of Piemonte Orientale (UPO), “Maggiore della Carità” University Hospital, Novara, Italy; Nephrology, Dialysis and Kidney Transplant Unit, IRCCS Azienda Ospedaliero-Universitaria di Bologna, Bologna, Italy; Nephro-Urological Department, AUSL “Toscana Sud Est”, Italy; Nephrology Unit, ASST Nord Milano, Ospedale Bassini, Cinisello Balsamo, Italy; Unit of Nephrology, Dialysis and Kidney Transplant. Civico Hospital of Palermo, Palermo, Italy; Internal Medicine, Department of Clinical and Experimental Medicine, University of Messina, Messina, Italy; Nephrology and Dialysis Unit, “Giovanni Paolo II” Hospital, Sciacca, Italy; Nephrology and Dialysis Unit, Department of Medical Specialties, ASLCN1, Cuneo, Italy; Division of Nephrology and Dialysis, “Miulli” General Hospital Acquaviva delle Fonti, Italy; Division of Nephrology and Dialysis, Department of Medicine; Santa Maria Goretti Hospital, Latina, Italy; Nephrology Unit, Hospital V. Fazzi, Lecce, Italy; Nephrology and Dialysis Unit, Hospital Santa Maria della Pietà, Nola, Italy; Nephrology Unit, AORN dei Colli, Naples, Italy; Nephrology and Dialysis Unit, Department of Clinic and Experimental Medicine, University of Messina, Messina, Italy; Nephrology and Dialysis Unit, “Guglielmo da Saliceto” Hospital, Piacenza, Italy; Nephrology and Dialysis Unit, County Hospital of Macerata, Macerata, Italy; Nephrology Unit, Hospital A. Fiorini, Terracina, Italy; Nephrology and Dialysis Unit, Department of Advanced Medical and Surgical Sciences, University of Campania, Naples, Italy

**Keywords:** albuminuria, CKD, dapagliflozin, real world, SGLT2 inhibitors

## Abstract

**Background:**

Sodium-glucose co-transporter-2 inhibitors (SGLT2i) are recommended for reducing the renal and cardiovascular risk in patients with chronic kidney disease (CKD) based on the positive results reported by clinical trials. However, real-world data on the efficacy and the safety of these drugs in CKD population followed in nephrology setting are lacking.

**Methods:**

We report the effects of dapagliflozin in CKD patients by using data collected during a learning program in which 105 nephrologists added dapagliflozin (10 mg/day) to consecutive patients referred to their renal clinics. Efficacy endpoints were the albuminuria change and the determinants of an albuminuria decline ≥30%. Adverse events were also collected.

**Results:**

A total of 1724 patients with CKD (age 67.4 ± 13.2 years, 72.8% males, diabetes 59.9%, eGFR 43.5 ± 17.4 ml/min/1.73 m^2^, severe albuminuria 70.1%) received dapagliflozin for 4 ± 1 months. Dapagliflozin significantly reduced body weight (−1.3 kg), eGFR (−0.27 ml/min/month), and blood pressure (−3.6/−1.7 mmHg). Albuminuria declined by 25.1% (95%CI 23.0–27.2) from 500 mg/day [IQR 225–1425] to 320 mg/day [IQR 100–900]. Albuminuria reduction was ≥30% in 48.3% of patients, 0–29% in 37.6% while it increased in 14.1% of patients. At logistic regression analysis, older age, female sex, use of mineralocorticoid receptor antagonist, higher eGFR, and higher albuminuria were all significant predictors of albuminuria decline ≥30%. We collected 46 side effects leading to drug discontinuation in 36 patients (2%), with acute kidney injury and urinary tract infection being the most frequent adverse events.

**Conclusions:**

We provide evidence of the anti-proteinuric efficacy of short-term dapagliflozin in the presence of good safety profile in patients with CKD followed in nephrology.

KEY LEARNING POINTS **What was known:**

- In patients with chronic kidney disease (CKD), treatment with dapagliflozin is associated with a significant improvement of cardio-renal outcome.
- Data supporting their use mainly derive from randomized clinical trials but real-world data on the efficacy and the safety of these drugs in CKD population followed in nephrology setting are lacking.

**This study adds:**

- In large cohort of patients with CKD followed in the real-word nephrology practice (*n* = 1724), dapagliflozin significantly declined albuminuria by 25%. A clinically relevant albuminuria reduction from baseline ≥30% occurred in 48.3% of patients.
- Older age, female sex, use of mineral-receptor antagonists, higher eGFR, and higher albuminuria were significant predictors of an effective anti-albuminuric response (≥30%) to dapagliflozin.
- In this real-world experience, adverse events of dapagliflozin leading to drug discontinuation occurred in 2% of patients.

**Potential impact:**

1. These data may reassure nephrologists on the efficacy and safety of SGLT2i in their everyday clinical practice and possibly contribute to abating the clinical inertia toward this class of drug that, despite the solid evidence on cardiovascular and renal protection, still remains largely under prescribed.

## INTRODUCTION

Randomized clinical trials (RCTs) have consistently demonstrated that in patients with non-dialysis chronic kidney disease (ND-CKD), sodium-glucose co-transporter-2 inhibitors (SGLT2i) are effective in reducing the cardiovascular risk and in retarding the progression of renal disease [1–4], thus substantially increasing the time spent free of dialysis [5]. Therefore, this drug class is now recommended as the first-line strategy for ND-CKD patients with and without diabetes with an evidence level even higher than renin–angiotensin system (RAS) inhibitors [6]. Unfortunately, despite the beneficial effects on health outcome and guideline recommendations, use of SGLT2i is disappointingly low among ND-CKD patients independent from diabetic status [7–11]. The potential explanations for clinical inertia on SGLT2i relate to low propensity to innovation, racial, ethnic, and socio-economic inequities, clinical conditions, and country-dependent healthcare policies that may limit the access to these medications [11–15]. In this regard, a global, prospective, observational study among ∼15 000 patients with diabetes mellitus from 37 countries highlighted a substantial variability in the use of SGLT2i according to the presence of comorbidities, with ND-CKD patients having 27% lower probability of receiving the prescription of these drugs [11]. Similarly, data from primary care in Canada showed that SGLT2i were underused and less likely to be prescribed to patients with pre-existing CKD with or without other comorbidities [16].

An additional barrier to the use of SGLT2i could be the limited information on efficacy and safety in the real world of daily clinical practice. This is an important issue when considering that the results of RCTs cannot be automatically translated into the clinical practice because demographic and clinical characteristics of patients recruited in the trials does not reproduce the picture seen in daily practice. Indeed, the generalizability of population enrolled in RCTs with SGLT2i is poor either in renal clinics (only 16.7% of patients potentially eligible for DAPA-CKD trial) [17] or in primary care (only 8.0% with same criteria of EMPA-KIDNEY trial) [18]. Therefore, providing real-world data on efficacy and safety of SGLT2i may “paradoxically” reinforce trial evidence and guidelines recommendations therefore favoring implementation of these drugs in clinical practice.

To date, no study has specifically addressed effectiveness of SGLT2i in CKD patients followed in the real-world setting of outpatient nephrology clinics. To fill this important gap of knowledge, we analyzed efficacy and safety data collected during an educational program for Italian nephrologists who started SGLT2i in patients with ND-CKD regularly followed in their renal clinics.

## MATERIALS AND METHODS

This paper reports a real-world data collection evaluating short-term effects of adding dapagliflozin in CKD patients. Data are collected during a three-phase learning program designed by the University of Campania, Naples, Italy aimed at improving adherence to clinical practice guidelines that started on April 2023. In the first phase, small groups of 10–15 nephrologists received educational material provided by the coordinating center (University of Campania) on the effects of SGLT2i in patients with CKD during local meetings coordinated by local facilitators. In the second phase, participating nephrologists were asked to report the data on the first use of dapagliflozin (the only drug of this class reimbursed in Italy for CKD patients with and without diabetes in 2023) in anonymized consecutive CKD patients referred to their renal clinics during a 4-month period. Each nephrologist was required to evaluate 15–20 patients by collecting data at baseline and after 3–6 months. Overall, 12 educational meetings including 105 clinicians practicing in 93 renal clinics were carried out across the country. Finally, in the third phase, results were object of discussion at closing local meetings. In this paper, we report the results of the output of this real-world experience. Since this data collection has educational purposes aimed at improving clinical practice, according to the Italian legislation, approval from the Ethical Committee is not required. Patients signed an informed consent for collecting data in anonymous form at the moment of enrollment.

The database included demographic, clinical history, physical examination with assessment of height, body weight, body mass index (BMI), blood pressure (BP), and medication profile and laboratory results (serum creatinine and potassium, albuminuria). Data, collected in anonymous electronic case reports, were subsequently sent to the coordinating center for quality assessment, storage, and analyses. Albuminuria was evaluated by 24 h urinary collection or as albumin/creatinine ratio according to local clinical practice; the two methods are considered equivalent for staging albuminuria categories [6]. GFR was estimated using the CKD-EPI equation. eGFR changes were estimated as ml/min/month to take into account the difference in timing of visit after baseline. BP was recorded in sitting position. Local investigators were asked to collect on voluntary basis data on albuminuria and eGFR measured in the visit performed 3–4 months before baseline (to confirm the steady state of these data at baseline). Adherence to the treatment was evaluated in each center according to local practice based on either glycosuria or by carefully reviewing the prescriptions for each patient at each visit. The lack of glycosuria or a missing rate of pill taking in the 2 weeks prior to any visit >20% were considered a measure of non-adherence to dapagliflozin prescription.

As a primary efficacy measure, we evaluated the percentage change from baseline of albuminuria. As secondary efficacy measures, we evaluated the determinants of the albuminuria decline of at least 30% and the incidence of regression in the albuminuria stage (from stage A3 to A1 or A2 and from stage A2 to A1). Drug-related adverse events reported by nephrologists were recorded to assess the safety of dapagliflozin in real-world clinical practice.

### Statistics

Continuous variables were reported as mean (SD), mean (95% confidence intervals), or median and interquartile [IQR] range based on their distribution. Comparisons between baseline data and follow-up visit were analyzed by paired Student's *t*-test or Wilcoxon test; the inter-group comparison was performed with ANOVA, an unpaired Student's *t*-test, or a Mann–Whitney test. Categorical variables (expressed as percentage) were analyzed using the Chi-square test or McNemar test. Pearson correlation analysis was used when appropriate.

Predictors of albuminuria decline from baseline ≥30% were estimated by multivariable logistic regression analysis with adjustment for the following baseline covariates identified *a priori*: older age (>70 years), sex, BMI, diabetes mellitus, history of cardiovascular disease (CVD), systolic BP, use of RAS inhibitors, use of mineral-receptor antagonists (MRA), eGFR, and albuminuria. Spironolactone and eplerenone were the only available MRA at the study time.

Data were analyzed using SPSS version 26 (IBM, Armonk, NY, USA).

## RESULTS

A total number of 2707 CKD patients were treated for the first time with dapagliflozin. Of these, 1762 had a follow-up visit and could be therefore included in the analysis (safety population). The efficacy population was restricted to 1724 patients, which are those who maintained dapagliflozin treatment over the entire follow-up period (Supplementary Fig. S1). Excluded patients (*n* = 945) did not differ from those included in the analysis (safety population), except for a higher eGFR, lower albuminuria, and lower prevalence of diabetes (Supplementary Table S1). Included patients were predominantly men (72.8%) and 48.2% of patients were older than 70 years. Diabetes was reported in 60.0% of patients, 43.6% had a previous CV event, and 25.2% were obese (BMI >30 kg/m^2^) (Supplementary Table S1).

### Efficacy population

At baseline, most patients included in the efficacy population had CKD stage 3–4 (85.1%) and moderate to severe albuminuria (92.8%) (Table 1). As reported in Table 1, at the follow-up visit performed on average after 4.1 ± 1.4 months, dapagliflozin induced a significant reduction of systolic BP (by 3.6 mmHg, 95%CI 2.9–4.2) and diastolic BP (by 1.7 mmHg, 95%CI 1.3–2.2); the achievement of a BP goal of <130/80 mmHg increased from 23.7% to 30.8% (*P* < .001). The better BP control occurred despite a lower use of antihypertensive drugs (Table 1). Body weight decreased on average by 1.28 kg (95%CI 1.10–1.45) with 52.5% of the population showing a decrease ≥1.0 kg. The reduction in body weight was greater in patients receiving diuretics (1.59 kg, 95%CI 1.33–1.85) in comparison with those not receiving diuretics (0.97 kg, 95%CI 0.73–1.20, *P* = .001). Serum potassium level declined by 0.05 mEq/l (95%CI 0.03–0.08) as well as the prevalence of hyperkalemia (>5.5 mEq/l) from 3.4% to 1.6% (*P* < .001); these changes occurred in the presence of similar prescription rate of potassium binders (0.9% and 1.2% in the two visits, respectively *P* = .109).

**Table 1:** Changes of clinical and laboratory parameters in patients receiving dapagliflozin (efficacy population, *n* = 1724).

	Baseline	Follow-up visit	*P*
Body weight (kg)	79.6 ± 15.4	78.4 ± 14.8	<.001
eGFR (ml/min/1.73 m^2^)	43.5 ± 17.4	42.5 ± 17.7	<.001
eGFR stage (%)			<.001
G1–G2	14.6	14.5	
G3a	22.4	20.4	
G3b	42.2	40.8	
G4	20.8	24.3	
Albuminuria (mg/24 h)	500 [225–1425]	320 [100–900]	<.001
Albuminuria stages (%)			<.001
A1	7.2	12.6	
A2	22.7	32.4	
A3	70.1	55.0	
Serum potassium (mEq/l)	4.56 ± 0.51	4.50 ± 0.48	<.001
Systolic BP (mmHg)	132 ± 16	129 ± 14	<.001
Diastolic BP (mmHg)	77 ± 10	75 ± 9	<.001
Antihypertensive drugs (*N*)	2.32 ± 1.14	2.07 ± 1.21	<.001
RAS inhibitors (%)	76.2	65.8	<.001
Diuretics (%)	49.5	43.3	<.001
Calcium channel blockers (%)	43.2	41.0	.005
Beta blockers (%)	50.6	48.2	.001
MRA (%)	6.8	6.3	.211

Data are mean (SD), median [IQR] or percentage.

**Figure 1:**
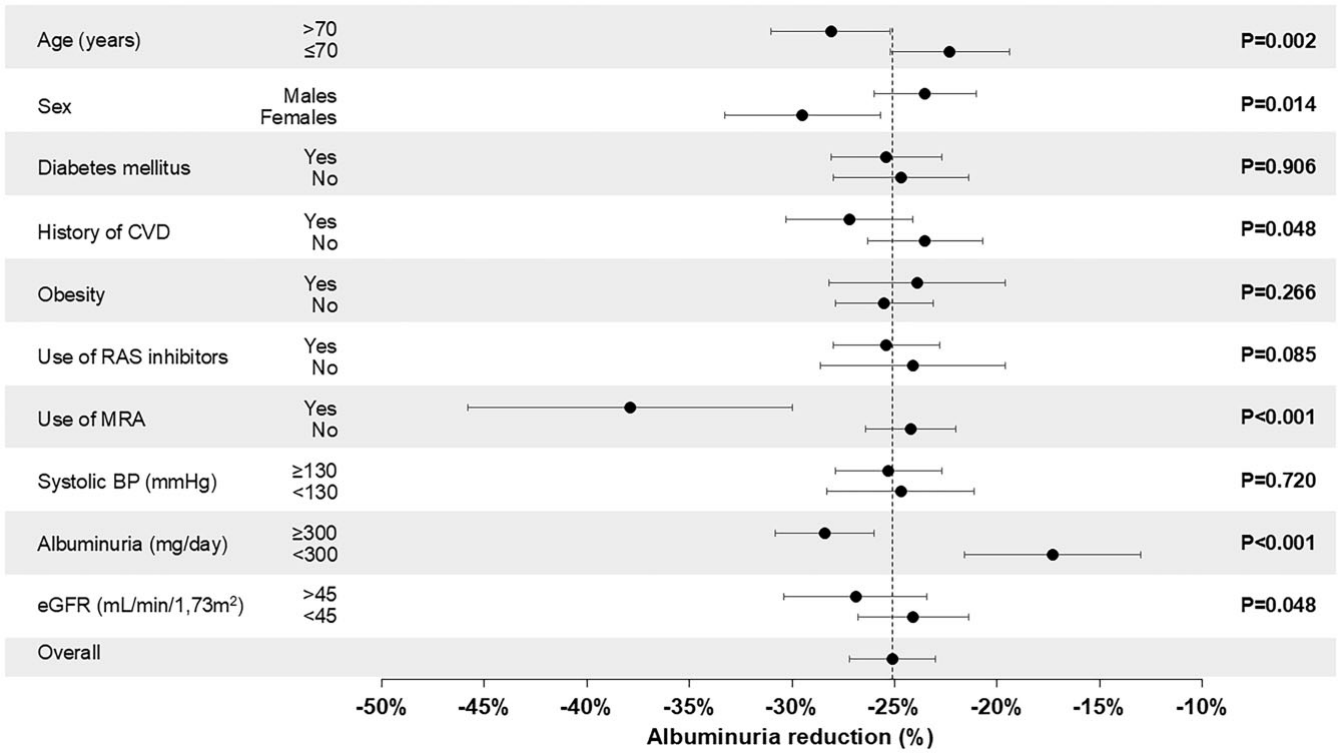
Changes from baseline of albuminuria in the whole population and after stratification for comorbidities, renal function, albuminuria, and treatment.

Albuminuria declined by 25.1% (95%CI 23.0–27.2). The prevalence of moderate to severe albuminuria was significantly reduced from 92.8% to 87.4%, with a regression in albuminuria stage occurring in 346/1724 patients (20.1%). In particular, among patients with albuminuria in stage A3 (*n* = 1208), regression of albuminuria was detected in 23.2% (20.6% to stage A2 and 2.6% to stage A1) while among those in albuminuria stage A2 (*n* = 391), 16.9% moved to stage A1. As reported in Fig. 1, albuminuria decline was greater in older patients (>70 years), in women, in patients with eGFR >45 ml/min/1.73 m^2^, in patients with a history of CVD, and in patients receiving MRA; conversely, it did not differ according to the presence of diabetes, obesity, controlled systolic BP, and the use of RAS inhibitors. No correlation was found between albuminuria reduction and change in body weight (*r* = 0.004, *P* = .855), eGFR (*r* = 0.018, *P* = .450), or duration of follow-up (*r* = 0.014, *P* = .558). In the whole population, a decline of albuminuria ≥30% was detected in 48.3% of patients (in 21.6% of patients albuminuria delcine was 30–50% and in 26.7% albuminuria declined more than 50%). We also found a suboptimal albuminuria decline (0%–29%) in 37.6% of patients and an increase of albuminuria in 14.1% of patients. When patients were stratified according to the entity of anti-albuminuric response (Table 2), we found that patients with a greater albuminuria decline were older, had a history of CVD, had higher eGFR, had lower BP, and used MRA more frequently. At multivariable logistic regression analysis, we found that an age >70 years, female sex, use of MRA, higher eGFR, and higher albuminuria were all basal predictors of an albuminuria decline ≥30% after starting dapagliflozin (Table 3).

**Table 2:** Baseline clinical characteristics in efficacy population overall and stratified by entity of albuminuria change.

		Stratified by albuminuria change				
	Overall	Any increase	Decline by 0%–29%	Decline by 30%–50%	Decline by >50%	*P*
*n* (%)	1724	243 (14.1%)	649 (37.6%)	372 (21.6%)	460 (26.7%)	
Age (years)	67.4 ± 13.2	65.6 ± 13.5	68.1 ± 12.7	65.3 ± 13.8	69.0 ± 12.7	<.001
Males (%)	72.8	77.8	74.1	70.4	70.2	.102
BMI (kg/m^2^)	27.6 ± 4.9	27.8 ± 4.8	27.6 ± 5.0	27.7 ± 4.7	27.4 ± 5.0	.646
Obesity (%)	25.1	27.2	26.0	24.5	23.0	.579
Diabetes mellitus (%)	59.9	56.0	62.4	58.3	59.6	.301
History of CVD (%)	43.6	40.3	42.4	43.5	47.0	.313
eGFR (ml/min/1.73 m^2^)	43.5 ± 17.4	44.1 ± 17.6	41.5 ± 16.2	44.6 ± 17.8	45.3 ± 18.2	.001
Albuminuria (mg/24 h)	500 [225–1425]	420 [178–1119]	380 [146–1200]	800 [300–1600]	556 [300–1500]	<.001
Systolic BP (mmHg)	132 ± 16	132 ± 16	132 ± 16	135 ± 16	131 ± 16	.008
Diastolic BP (mmHg)	77 ± 10	77 ± 10	76 ± 10	78 ± 9	76 ± 10	.038
BP target (%)	24.0	21.4	24.8	21.2	26.3	.258
BP drugs (*N*)	2.32 ± 1.14	2.33 ± 1.08	2.29 ± 1.15	2.34 ± 1.16	2.35 ± 1.16	.885
RAS inhibitors (%)	76.2	77.0	73.2	80.6	76.3	.042
MRA (%)	6.8	4.1	5.5	6.2	10.4	.003
Diuretics (%)	49.5	50.2	47.0	47.0	54.8	.052
Follow-up (months)	4.1 ± 1.4	4.2 ± 1.5	4.0 ± 1.3	4.0 ± 1.5	4.1 ± 1.6	.240

Data are mean (SD) or median [IQR].

**Table 3:** Baseline predictors of albuminuria reduction >30% in patients receiving dapagliflozin (efficacy population, *n* = 1724).

	Odds ratio	95%CI	*P*
Older age (>70 years)	**1.25**	**1.01–1.54**	**.039**
Females (vs males)	**1.38**	**1.11–1.72**	**.004**
BMI (kg/m^2^);	0.99	0.97–1.01	.402
Systolic BP (5 mmHg)	1.02	0.98–1.05	.313
Diabetes mellitus (yes vs no)	0.93	0.73–1.12	.345
History of CVD (yes vs no)	1.21	0.98–1.50	.079
Use of RAS inhibitors (yes vs no)	1.16	0.92–1.47	.210
Use of MRA (yes vs no)	**1.56**	**1.04–2.34**	**.031**
eGFR (10 ml/min/1.73 m^2^)	**1.14**	**1.07–1.21**	**<.001**
Albuminuria (g/day)	**1.17**	**1.08–1.27**	**<.001**

In bold are indicated significant predictors.

During the observation period, eGFR declined on average by 0.27 ml/min/month (95%CI 0.14–0.39); in particular, eGFR was reduced at follow-up visit in 53.0% of patients, with only 4.1% who experienced an eGFR decline >30%, while eGFR was stable in 12.7% and increased in 34.3% of patients. The decline in eGFR was significantly greater in non-diabetic patients, in patients free of CVD, in those with more preserved renal function (eGFR >45 ml/min/1.73 m^2^), and in patients with severe albuminuria (Supplementary Fig. S2). The entity of eGFR decline did not differ according to diuretic use (*P* = .306). In a subgroup of 833 patients, we also collected albuminuria and eGFR in the last visit before baseline (3 months); the two parameters did not differ between the two visits (pre-baseline and baseline) (Supplementary Table S2).

### Safety population

During the follow-up, among the 1762 patients included in the safety population, nephrologists collected 46 side effects that led to drug discontinuation in 36 patients (Table 4); withdrawal of dapagliflozin did not differ between older and younger patients (53% and 47%, respectively, *P* = .83). Two more patients stopped dapagliflozin treatment due to pregnancy and COVID-19 infection, respectively.

**Table 4:** Adverse events leading to dapagliflozin discontinuation (46 events in 36 patients).

Cause	Number of events	Incidence rate (*n*/100 patient-years)
Urinary tract infections	10	1.70
Acute kidney injury	9	1.53
Worsening of renal function	4	0.68
Urinary symptoms	4	0.68
Volume depletion	3	0.51
Symptomatic hypotension	3	0.51
Pruritus	2	0.34
Intolerance	1	0.17
Malaise	1	0.17
Headache	1	0.17
Hyperammonemia	1	0.17
Loss of appetite	1	0.17
Vomiting	1	0.17
Hyperpyrexia	1	0.17
Gastric burn	1	0.17
Start of immunosuppressive treatment	1	0.17
Planned surgery	1	0.17
Suspected causal agent of interstitial nephritis	1	0.17

Overall, infections of the genito-urinary tract were observed in 10/1762 patients (0.6%). AKI occurred in 25/1762 patients (4.2/100 patient-years); among these, nine patients stopped dapagliflozin (five were hospitalized). Of the remaining 16 patients, 10 developed AKI that did not need either hospitalization or drug withdrawal and 6 patients were hospitalized without stopping dapagliflozin. We recorded 77 hospitalizations; of these, 23 occurred for cardiovascular reasons (myocardial infarction *n* = 5, coronary revascularization *n* = 5, acute heart failure *n* = 4, arrhythmia *n* = 4, peripheral vascular disease *n* = 3, mitral valve repair *n* = 1, and stroke *n* = 1), 13 for renal causes (AKI *n* = 11, hyperkalemia *n* = 1, starting chronic dialysis *n* = 1), 20 for surgery, 13 for infections, and 8 for other reasons.

## DISCUSSION

Our data extend to the clinical practice in nephrology the favorable effects on albuminuria evidenced in the setting of clinical trials [19]. Notably, the entity of anti-albuminuric response detected in our real-world patients (−25%) is slightly lower than that reported by DAPA-CKD at the second visit of the trial performed in month 4 (∼−40%). This difference may be ascribed to the higher basal albuminuria level in DAPA-CKD (ACR ∼950 mg/g), where the main inclusion criterion was albuminuria 200–5000 mg/g [2]. On the other hand, the response is more similar to the mean decrease detected in the EMPA-KIDNEY trial (−19%) where median albuminuria at baseline was ∼331 mg/g [3]. These data support the knowledge that the higher the basal albuminuria, the higher the extent of the anti-albuminuric response [20]. The correspondence of albuminuria values measured at baseline and 3 months before reinforces the anti-albuminuric response to dapagliflozin.

In comparison with seminal RCTs with SGLT2i in patients with ND-CKD [2, 3], the population enrolled in the present study is 3 to 6 years older, with more males and a prevalence of diabetes similar to the DAPA-CKD trial (68%) but higher than the EMPA-KIDNEY study (46%). In our patients, mean eGFR was similar to that reported in DAPA-CKD but 6 ml/min/1.73 m^2^ higher than EMPA-KIDNEY. Finally, an important difference from previous RCTs is the presence of a substantial number of patients not receiving RAS inhibitors (*n* = 411, 23.8%), which was an exclusion criterion for both DAPA-CKD and EMPA-KIDNEY studies, while being common in the real-world setting.

Regression of albuminuria in our short-term observation occurred in 23.2% of patients at stage A3, an incidence slightly higher than that detected in DAPA-CKD (16.5%) during the whole study period (2.4 years). It is interesting to note that albuminuria reduction ≥30% from baseline, now considered a valid surrogate of CKD progression [21], was detected in 48% of patients (Table 2). Baseline predictors of albuminuria reduction were older age, female sex, more preserved eGFR, higher proteinuria, and use of MRA (Table 3). It is important to underline the sex difference in the anti-proteinuric effect of dapagliflozin (−30% in women and −23% in men, *P* = .01) when considering that women are frequently undertreated with this new class of renoprotective drugs [12, 14, 22–24], as well as with RAS inhibitors [25]. Of similar relevance is the finding that the anti-albuminuric effect of dapagliflozin is also in very evident in older patients (Fig. 1 and Table 3). Indeed, these patients, usually underrepresented in RCTs [2, 3], are frequently left untreated with SGLT2i mainly because of safety concerns associated with frailty, concomitant drugs, and cognitive impairment [26].

In our study, no difference emerged in adverse events with age, according to a large pharmacovigilance study [27]. Previous RCTs evidenced that use of steroidal and non-steroidal MRA induced a significant reduction in albuminuria [28, 29]. This effect persisted when dapagliflozin was added to spironolactone or eplerenone, or when finerenone was added to SGLT2i [30, 31]. In a randomized crossover trial, Provenzano *et al.* demonstrated that combining eplerenone with dapagliflozin resulted in an anti-albuminuric effect (−53%) that was additive in comparison with the single drugs (−20% and −34% with dapagliflozin and eplerenone alone, respectively) [32]. We confirmed these observations in our real-world data; indeed, the anti-albuminuric effect was significantly greater when dapagliflozin was added in patients treated with traditional MRA vs those untreated (−37.8% vs −24.2%, respectively) (Fig. 1). Furthermore, patients treated with MRA had a 56% higher probability of reaching an optimal albuminuria reduction (Table 3). This result may support an additive effect of MRA-SGLT2i combination on albuminuria when considering that patients under combined treatment have similar albuminuria levels at baseline in comparison with patients not taking MRA.

An original finding of the present study relates to the anti-albuminuric efficacy of dapagliflozin in patients not receiving RAS inhibition. Indeed, these patients, not enrolled in previous RCTs [2, 3], displayed an albuminuria reduction (−24.1%) with dapagliflozin similar to that recorded when dapagliflozin was added on top of RAS inhibitors (−25.4%) (Fig. 1); in addition, at multivariate analysis, these drugs did not affect the probability of reaching the threshold of the 30% of decline in albuminuria (Table 3). A similar finding has been previously reported in a pooled analysis of phase-2 and phase-3 RCTs only in patients with diabetes in whom the changes of urinary albumin-creatinine ratio after 24 weeks of dapagliflozin were of the same magnitude in patients whether receiving RAS inhibitors or not [33].

Hemodynamic changes observed in our population replicated that reported in RCTs. Indeed, dapagliflozin reduced BP but the entity of pressor effect in our patients (−3.6/1.7 mmHg) is slightly higher than that reported in the DAPA-CKD (−2.9/1.0 mmHg) [34] and EMPA-KIDNEY trials (−2.6/−0.5 mmHg) [3]. However, our data were obtained in a shorter time and, therefore, we cannot exclude that this slightly larger BP decline in our study could be influenced by a carry-over effect of the acute BP decline usually seen in the first 2 weeks of dapagliflozin [34]. We also detected in our population a significant decline in body weight (−1.28 kg on average); not surprisingly, this reduction is not related to anti-albuminuric effect likely because dapagliflozin lowers body weight in part by reducing fat mass due to the negative calorie balance [35].

When considering renal function, we found a significant decline of eGFR that was significantly larger in patients with fewer comorbidities (diabetes and CVD) and in those with higher albuminuria and higher eGFR (Supplementary Fig. S2). In these patients, the greater drop of eGFR could be explained by a more frequent use of RAS inhibitors in these subgroups. Indeed, prescription rate of these drugs at the end of follow-up period was higher in patients without diabetes (73.5% vs 60.2% in patients with diabetes) or CVD (68.4% vs 62.5% in patients with CVD), with severe albuminuria (70.2% vs 54.4% in patients with albuminuria <300 mg/day), and with more preserved renal function (68.8% vs 63.3% in patients with eGFR<45 ml/min/1.73 m^2^). The combination of dapagliflozin and RAS inhibitors, by acting on both afferent and efferent glomerular arterioles, may produce a larger reduction in intra-glomerular pressure that becomes particularly evident in patients with higher glomerular hyperfiltration at baseline [36, 37]. However, in the whole population, eGFR did not differ in patients using RAS inhibitors or not (Fig. 1); this finding is in line with data from real-world clinical practice in patients with type 2 diabetes showing that the annual rate of eGFR change did not change with RAS inhibitor use [38]. It is important to note that it is difficult to compare the entity of eGFR slope in our population with other studies due to the relatively short time of our observation.

During the follow-up, only 2.0% patients stopped dapagliflozin treatment due to side effects; this result is hardly comparable with the higher discontinuation rate reported in RCTs (12.7%) [2], due to the different length of follow-up. The same occurred for the incidence of volume depletion (0.2% in our study as compared with 5.9% in the DAPA-CKD trial) [2]. AKI incidence seems to be higher in our study (4.2/100 patient-years) compared with DAPA-CKD (1.0–1.5/100 patient-years) [4]. This occurred despite participating nephrologists implementing mitigating strategies against AKI, as testified by a significant reduction in the number of prescribed BP drugs and a significant withdrawal of RAS inhibitors (occurring in 16.8% of patients receiving RAS inhibitors at baseline) and diuretics (occurring in 17.8% of patients receiving diuretic at baseline) (Table 1). It is likely that the lower incidence rate of AKI in DAPA-CKD trial is strongly influenced by the enrollment of a highly selected population, which is typically less representative of the population regularly followed in renal clinics [17].

The main limitation of this study is the observational design that does not allow proof of any cause–effect relationship. A further limitation relates to the duration of follow-up (4.1 months, on average) that allows evaluation of only the short-term anti-albuminuric effect of dapagliflozin. In addition, we focused data collection on albuminuria and main hemodynamic parameters while other laboratory data, useful for a complete assessment of the effect of dapagliflozin, were lacking. Finally, we could not ascertain the difference according to the underlying cause of renal disease, as this information was not included in our database. However, based on subgroup analyses of RCTs and metanalysis, it is now widely accepted that the protective effect of SGLT2i is independent from the primary kidney disease [2–4]. This finding has been further supported by observational data showing that SGLT2i effectively reduces proteinuria in the whole spectrum of glomerular diseases [39, 40]. Strengths of the present study are the evaluation of the efficacy and safety profile of dapagliflozin outside the setting of a clinical trial and specifically in a large cohort of patients with CKD attending renal clinics.

In conclusion, short-term dapagliflozin treatment significantly decreases albuminuria in a large cohort of patients regularly followed in renal clinics. The anti-proteinuric effect is more pronounced in patients with severe albuminuria and higher eGFR and independent of diabetic status, cardiovascular comorbidities, BP level, and concomitant therapies (RAS inhibitors and diuretics). The safety profile was not substantially different from that reported in RCTs.

Overall, these data may reassure nephrologists on the efficacy and safety of SGLT2i in their everyday clinical practice and possibly contribute to abating the clinical inertia toward this (not newer) class of drugs that, despite the solid evidence on cardiovascular and renal protection, still remains unacceptably high [7, 41].

## Supplementary Material

sfae396_Supplemental_File

## Data Availability

R.M. has full access to all the data in the study and takes responsibility for the integrity of the data and the accuracy of the data analysis. The datasets generated during and/or analyzed in the current study are available from the corresponding author upon reasonable request.

## Notes

Each author contributed important intellectual content during manuscript drafting or revision and agrees to be personally accountable for the individual's own contributions and to ensure that questions pertaining to the accuracy or integrity of any portion of the work, even one in which the author was not directly involved, are appropriately investigated, and resolved, including with documentation in the literature if appropriate.
