# Supplementary material for: Efficacy and safety of dapagliflozin in patients with CKD: real-world experience in 93 Italian renal clinics
Source: Clin Kidney J. 2024 Dec 3;18(1):sfae396. doi: 10.1093/ckj/sfae396 (PMC11744308; doi:10.1093/ckj/sfae396)
Supplement: sfae396_Supplemental_File [file sfae396_supplemental_file.pdf]

Supplementary material for the manuscript:

EFFICACY AND SAFETY OF DAPAGLIFLOZIN IN PATIENTS WITH CKD: REAL WORLD EXPERIENCE IN 93 ITALIAN RENAL CLINICS

List of investigators

Claudio Americo (Ospedale di Forlì), Francesca Apponi, Luigi Turchetta (ASL Frosinone), Caterina Oriana Aragona, Guido Gembillo, Antonino Ragusa (AOU Gaetano Martino, Messina), Silvana Baranello (Ospedale Antonio Cardarelli di Campobasso), Sara Barbieri, Federica Tomatis (ASL CN2, Alba – Bra, Cuneo), Simona Barbuto, Matteo Righini (Policlinico S. Orsola Malpighi, Bologna), Riccardo Barisonzo (Ospedale di Bolzano), Sara Beati (Ospedale di Versilia, Viareggio), Mirco Belingheri (Fondazione IRCCS Ca' Granda Ospedale Maggiore Policlinico di Milano), Marianna Bencivenga, Gabriella Di Natale (P.O. dei Pellegrini ASL NA 1 Centro, Napoli), Eleonora Bernabei (Ospedale S. Eugenio, Roma), Beatrice Braccagni (Ospedale di Pisa), Laura Bregoli (ASST Spedali Civili di Brescia, Presidio di Montichiari), Daniele Cagna (ASST Valtellina e Alto Lario, Presidio di Sondrio), Cristina Calvi (A.O.N. “SS. Antonio e Biagio e Cesare Arrigo”, Alessandria) Martina Camplese (ASL di Teramo), Rossella Cannavò (Ospedale Santa Maria Annunziata di Bagno a Ripoli), Valeria Cassini (ASP di Sciacca), Giusy Chiarelli (ASST di Cremona), Angela Cicciarelli (P. O. Fabrizio Spaziani, Frosinone), Salvatore Coppola (P.O. di Piedimonte Matese), Alessia Corvo (Ospedale di San Bonifacio), Fatma Cossetti (P. O. Santo Spirito in Sassia, Roma), Fabrizio Cristiano, Elsa Marisi (Ospedale “San Pio di Pietrelcina”, Vasto), Daniele Damiani (ASL TO 4, Torino), Sara De Amicis (AUSL di Piacenza), Giuseppina D'Ettorre (Ospedale SS. Annunziata di Taranto), Carlo Di Benedetto (ASL AT, Asti), Diana Teodora Dodoi (ASL TO5, Torino), Valentina Donato (ASST Valcamonica), Samantha Esposito (USL9 Siena), Efstratios Fasianos (Ospedale della Murgia Fabio Perinei, Altamura), Antonio Federico (Ospedale “Carlo Urbani “Jesi), Annamaria Ferri (ISS San Marino), Luca Fraizzoli (Ospedale San Pellegrino di Castiglione delle Stiviere), Alberto Garrone (Nuovo Ospedale degli Infermi, Biella), Giuseppe Gernone (Ospedale S. Maria degli Angeli, Putignano), Elisa Giglio (ASP di Ragusa), Gelsomina Giordano (Ospedale di San Felice a Cancello), Filippo Girasole (P.O. di Patti), Rosa Grimaldi, Daniela Mastroluca, Paolo Francesco Steri (ASL di Latina), Gabriele Guglielmetti (Università del Piemonte Orientale "Amedeo Avogadro", Novara), Raffaella Guido (A.O.U. Consorziale Policlinico di Bari), Valentina Iacono (Azienda ULSS 5 Polesana, Rovigo), Antonietta La Verde (A.O.R.N. Sant’Anna e San Sebastiano, Caserta), Luigi Lattuca (ASP 9 di Trapani), Paolo Lentini (Ospedale di Bassano del Grappa), Chiara Maria Maggioni, Francesco Perna (ASST Sette Laghi, Varese), Anna Malinverno (Ospedale Sant'Anna di Como), Giovanni Manca Rizza (Ospedale di Pontedera), Loredana Manfreda, Salvatore Ruosi (ASL di Caserta), Elisabetta Manno (Ospedale Generale Regionale 'F. Miulli', Bari), Francesca Masselli (Ospedale Annunziata, Cosenza), Florjan Mehmeti (ASST di Melegnano e della Martesana), Alessandra Mele (P.O. di Pozzuoli), David Micarelli (Ospedale di Tivoli), Gaetano Montalto (P.O. di Taormina), Paola De Giovanni, Marcello Montevecchi (Ospedale degli Infermi di Rimini), Vincenzo Montinaro (Ospedale “Miulli” di Acquaviva delle Fonti), Maria Stella Musumeci (Ospedale Maria Paternò Arezzo, Ragusa),

Francesco Napolitano, Addolorata Porcelluzzi (P.O. "Dimiccoli", Barletta), Alessandro Naticchia (Pia Fondazione Panico, Tricase), Valentina Nicosia (Ospedale di Formia), Vincenzo Origlia (P.O. Mater Dei, Bari), Giovanni Otranto (Ospedale di Colleferro), Marco Palladino (ASL Toscana Sud Est, Grosseto), Francesco Pavese (P.O. di Castellammare Di Stabia), Paolo Perosa (Ospedale Agnelli, Pinerolo), Angelo Pica (Azienda Provinciale per i Servizi Sanitari Provincia Autonoma di Trento), Carmelo Adriano Piluso (Ospedale di Pistoia), Luca Piscitani P.O. di L'Aquila), Rosaria Polci (Ospedale C. e G. Mazzoni, Ascoli Piceno), Francesco Principe (NephroCare, Mirabella Eclano), Monica Racanati (P.O. di Venere, Bari), Francesca Radice (ASST Nord Milano), Roberta Renzulli (AORN Moscati, Avellino), Gennaro Santorelli (P.O. di Desio e Vimercate), Raffaele Scigliano (P.O. Monaldi, Napoli), Sara Signorotti (Ospedale M. Bufalini, Cesena), Giorgio Soragna (A. O. Ordine Mauriziano, Torino), David Sorbo (ULSS 8 Veneto, Vicenza), Federica Spaltini (Istituto Universitario di Studi Superiori di Pavia), Emiliano Staffolani (A.O. San Camillo Forlanini, Roma), Andrea Stucchi (ASST Santi Paolo e Carlo, Milano), Alice Tarroni (ASL 2 Liguria, Genova), Michela Tedesco (ASST Fatebenefratelli Sacco, Milano), Giuliana Tognarelli (ASL TO3, Torino), Maria Giovanna Vario (AOU Policlinico Paolo Giaccone, Palermo), Luigi Vecchi (A.O. Santa Maria, Terni), Fabio Violi (Ospedale Santa Scolastica, Cassino), Luca Visconti (Ospedali Riuniti Villa Sofia Cervello, Palermo), Tewoldemedhn Yabarek (P.O. di Manerbio), Maddalena Zambelli (USL di Imola).

Supplementary figure 1. Flow chart of the study.

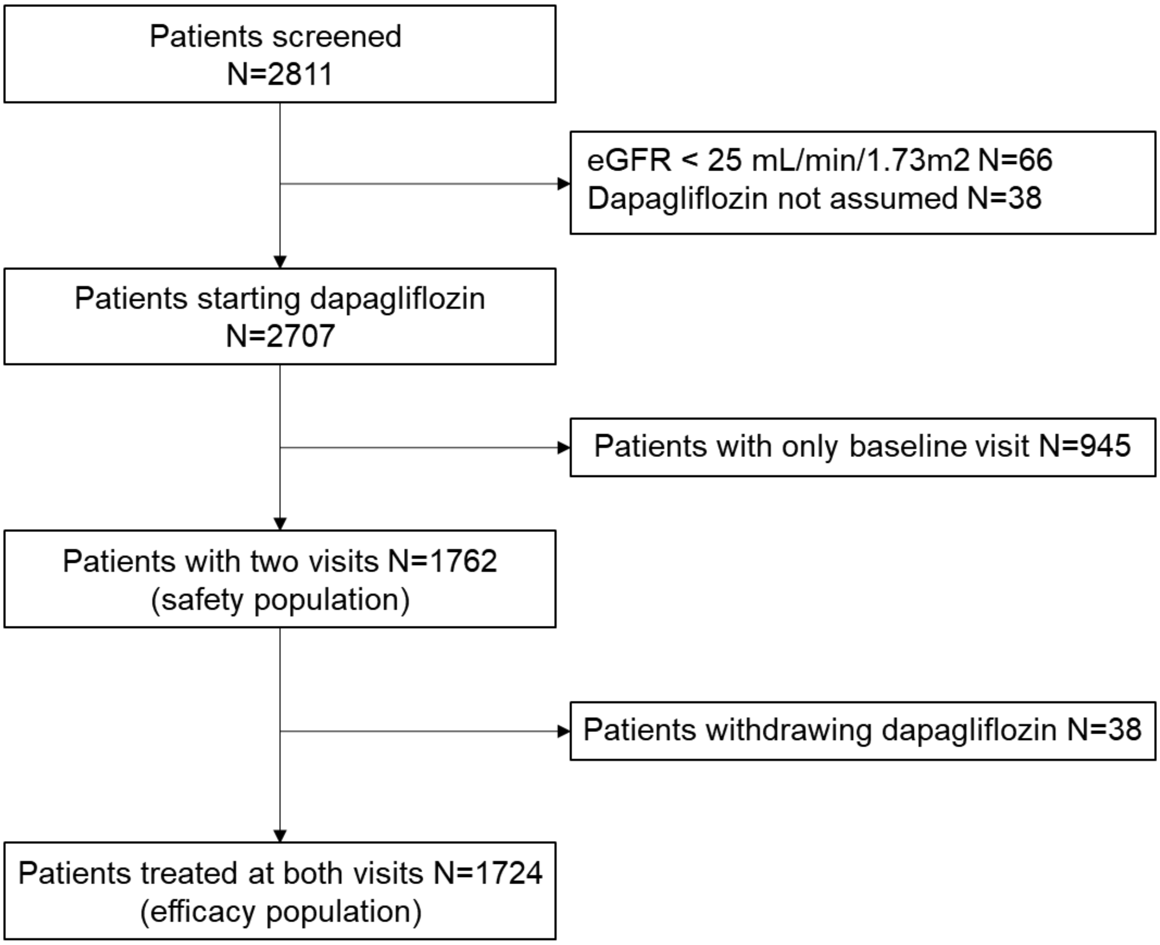

Supplementary Figure 2. Changes from baseline of eGFR in the whole population and after stratification for comorbidities, renal function, albuminuria and treatment.

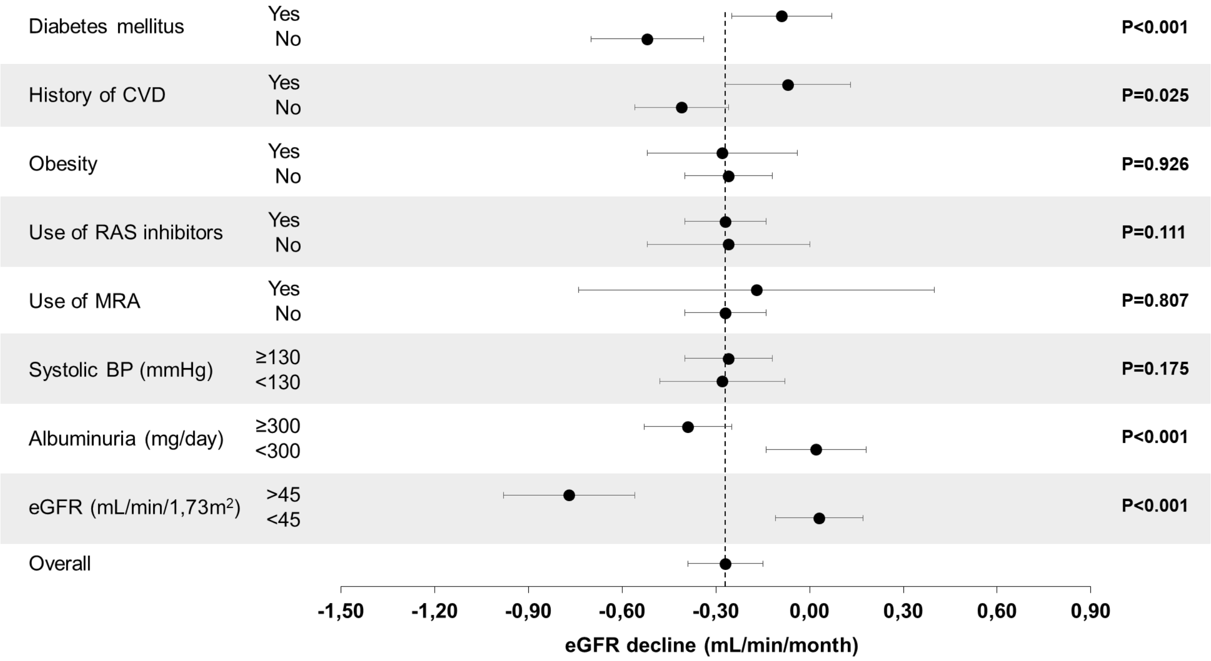

Abbreviations: CVD, cardiovascular disease; RAS, renin-angiotensin system; MRA, mineral-receptor antagonists; BP, blood pressure, eGFR estimated glomerular filtration rate.

Supplementary Table 1. Baseline clinical characteristics in included and excluded patients.

	**Included patients (N=1762)**	**Excluded patients (N=945)**	**P**
Age (years)	67.4±13.2	67.1±13.2	0.587
Males (%)	72.8	72.9	0.933
BMI (kg/m^2)	27.6±4.9	27.7±4.7	0.766
Diabetes (%)	60.0	51.9	<0.001
History of CVD (%)	43.6	42.1	0.445
eGFR (mL/min/1.73m^2)	43.5±17.4	46.2±16.4	<0.001
Albuminuria (mg/24h)	500 [220-1430]	364 [200-860]	<0.001
Systolic BP (mmHg)	133±16	134±16	0.102
Diastolic BP (mmHg)	77±10	77±10	0.845
Use of RAS inhibitors (%)	75.9	78.8	0.082

Supplementary Table 2. eGFR and albuminuria before and after adding dapagliflozin in the subset of 833 patients with available data.

	**Pre-DAPA**	**Baseline**	**Post-DAPA**	**P**
eGFR (mL/min/1.73m^2)	44.9±18.4	44.2±18.3	42.6±18.0	<0.001
Albuminuria (mg/24h)	500 [279-1365]	500 [200-1400]	340 [100-850]	<0.001
Change from Pre-DAPA (%)	-	5.5 (1.5 to 9.6)	-23.8 (-27.0 to -20.7)	-
Change from baseline (%)	-	-	-21.3 (-24.4 to -18.3)	-
